# Prevalence of *hepatitis B infection* and its associated factors among pregnant mothers attending antenatal care at public hospitals at Hararghe, Eastern Ethiopia

**DOI:** 10.3389/fgwh.2023.1056488

**Published:** 2023-04-27

**Authors:** Abbas Umer, Zelalem Teklemariam, Firayad Ayele, Melkamu Merid Mengesha

**Affiliations:** ^1^ West Hararghe Zone Health Office, Chiro, Ethiopia; ^2^ School of Medical Laboratory Sciences, Haramaya University College of Health and Medical Sciences, Harar, Ethiopia; ^3^ School of Public Health, College of Medicine and Health Sciences, Arba Minch University, Arba Minch, Ethiopia

**Keywords:** hepatitis B virus infection, prevalence, pregnant mothers, antenatal care, western Hararghe, eastern Ethiopia

## Abstract

**Background:**

Hepatitis B infection is one of the world's most serious public health problems, causing significant morbidity and mortality. More than 2 billion individuals around the world have been infected with the hepatitis B virus (HBV), and approximately 400 million people are chronically infected with the virus, with more than a million dying each year from hepatitis B virus-related liver disease. A newborn infant whose mother is positive for both HBsAg and HBeAg has a 90% chance of developing chronic infection by the age of 6. Its infectivity is a 100 times that of the human immunodeficiency virus, but it receives little attention in public health. Therefore, this study was conducted to assess the prevalence of *hepatitis B infection* and its associated factors among pregnant mothers attending antenatal care at public hospitals in west Hararghe, eastern Ethiopia 2020.

**Method:**

This institution-based cross-sectional study was conducted on 300 pregnant mothers selected by systematic random sampling from September to December 2020. Data were collected by face-to-face interview using a pretested structured questionnaire. A blood sample was collected and tested for *hepatitis B* surface antigen using the enzyme-linked immunosorbent assay test method. Data were entered into EpiData version 3.1 and exported to Statistical Package for the Social Science version 22 for analysis. Bivariate and multivariable logistic regressions were used to assess the association between outcome and predictor variables. *P*-value <0.05 was considered to be statistically significant.

**Results:**

The overall seroprevalence of *hepatitis B virus* infection was 8% [95% confidence interval (CI): 5.3–11.0] among pregnant mothers. History of tonsillectomy [adjusted odd ratio (AOR) = 5.7; 95% CI: 1.3–23.9], tattoo (AOR = 4.3; 95% CI: 1.1, 17.0), having multiple sexual partners (AOR = 10.8; 95% CI: 2.5, 45.9), and history of contact with jaundiced patients (AOR = 5.6; 95% CI: 1.2, 25.7) were factors associated with the seroprevalence of hepatitis B virus infection among pregnant mothers.

**Conclusion:**

The hepatitis B virus was highly prevalent. A history of tonsillectomy, tattooing, having multiple partners, and contact with jaundiced patients were factors associated with hepatitis B virus infection. To reduce HBV transmissions, the government should increase HBV vaccination coverage. All newborns should receive the hepatitis B vaccine as soon as possible after birth. It is also recommended that all pregnant women have HBsAg testing and antiviral prophylaxis to reduce the risk of transmission from mother to child. Hospitals, districts, regional health bureaus, and medical professionals should also educate pregnant women about hepatitis B virus transmission and prevention, both in the hospital and in the community, with a focus on modifiable risk factors.

## Introduction

*Hepatitis B* virus (HBV) is one among the prominent public health problems globally (1). The disease is caused by the hepatotropic deoxyribonucleic acid (DNA) virus and happens over the immune-mediated killing of diseased liver chambers (2, 3). *Hepatitis B* virus is a potentially life-threatening pathological virus of the liver that leads to critical or prolonged hepatitis (3). In childhood, less than 5% of infections result in chronic hepatitis (4).

Hepatitis B virus can cause coagulation defects, postpartum hemorrhage, organ failure, and high maternal mortality, as well as poor newborn outcomes such as stillbirth, neonatal deaths, severe, and prolonged liver disease. Early intervention and prevention of these illnesses is now a priority, with universal screening in antenatal care (ANC) and as part of reproductive health programs (3). It has an average incubation period of 75 days and can be detected in the blood within 30–60 days (3). In light of this, the virus is more contagious and powerful than the human immunodeficiency virus (HIV) virus (5, 6).

The World Health Organization (WHO) estimates that approximately 296 million people had chronic hepatitis B infection in 2019, with 1.5 million new infections occurring each year. In 2015, 2.7 million people were HIV coinfected (7) with a total of 68% of infected people in Africa and Western Pacific Asia (8). Furthermore, 5%–15% of populations in developing and poor-income countries are chronic carriers of hepatitis B infection (9). Furthermore, the World Health Organization reported that hepatitis is responsible for 1.34 million deaths, which is equivalent to deaths due to tuberculosis but higher than deaths due to HIV in 2015 (3). In addition, 1.8 million children less than 5 years are living with *hepatitis B virus* infection (10). Due to the high price of the vaccine, private access is less likely (11).

As a part of the sub-Saharan region, Ethiopia ranked medium to high endemicity for *HBV* infection (12). In Ethiopia, even if there is a lack of data representing the spread of *HBV* infection nationally, according to the findings of some individual studies, the country is regarded as having a high burden of the disease (12). Systematic reviews and meta-analyses revealed that the prevalence of HBV in the general population ranged from 6% to 7.4% (13) whereas 5%–7% of pregnant mothers were a major source of disease for newborns (14).

Despite the fact that the disease is endemic in all populations and/or pregnant mothers in the country, there has been no research done in west Hararghe, eastern Ethiopia, where the prevalence and factors associated with HBV infection among pregnant mothers are unknown. Furthermore, evidence-based information on the prevalence and risk factors for HBV infection in pregnant women enables effective HBV prevention and management. Therefore, the present study is aimed to estimate the seroprevalence and to identify factors associated with *hepatitis B virus* infection among pregnant mothers attending public hospitals in west Hararghe, Ethiopia.

## Materials and methods

### Study area and study period

The study was conducted in the west Hararghe zone of the Oromia region from September to December 2020. West Hararghe is one of the 21 administrative zones in the Oromia regional state. Chiro town, which is 317 km to the east of Addis Ababa, serves as the zone's capital. Based on information from the 2007 Ethiopian CSA census, it is estimated that there are 2,667,000 people living in the west Hararghe zone. There are 17 woredas and 496 kebeles that make up the administrative division of the zone, of which 39 are urban and 457 are rural. In the zone, there are currently 4 public hospitals, 85 health centers, and 465 health posts. During the months of September to December 2020, the study was carried out at the public hospitals in Gelemso and Chiro (Source: 2019–2020 document profile from the West Hararghe Zonal Health Office).

### Study design and population

A cross-sectional study design was conducted among pregnant mothers attending ANC clinics at public hospitals in west Hararghe. All confirmed pregnant mothers who were chosen through a systematic random sampling method and gave their written consent to take part in the study during the study period were included. Those who were mentally unconscious or who made a second ANC visit during the data collection period were excluded.

### Sample size, sampling technique, and procedure

The sample size of the study was determined using a single population proportion formula by considering the prevalence of *HBV infection* in pregnant mothers at the Deder hospital in eastern Ethiopia (15), with a prevalence of 6.9%, a 95% confidence level (CI), and a margin of allowable error of 3%. To minimize the errors arising from the likelihood of noncompliance, 10% of the sample size was added. The final sample size was 302. Systematic random sampling was used until the required sample was reached.

**Figure 1:**
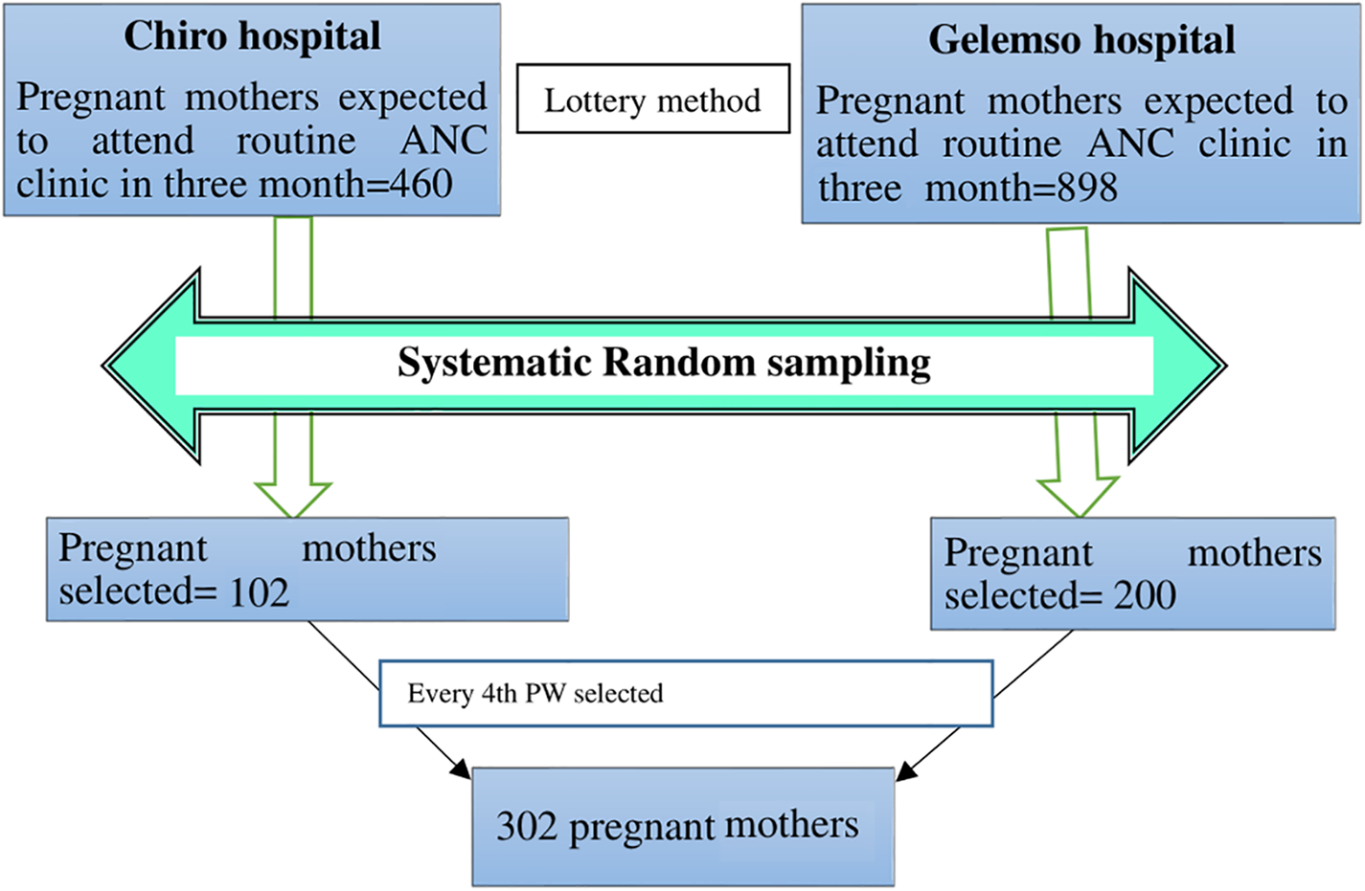
Schematic presentation of the sampling procedure and sampling technique for including in the study pregnant mothers attending routine antenatal clinics in west Hararghe public hospitals, 2020.

A total of 1,358 pregnant mothers were expected to attend ANC clinics in two hospitals in the west Hararghe zone (460 pregnant mothers at the Chiro General Hospital and 898 pregnant mothers at the Gelemso General Hospital) for routine ANC from a previous registered annual number of clients served. A total of 302 study participants were selected proportionally from each hospital. A systematic random sampling technique was applied to select study participants. We took 3 months' average sampled population from the registration books, which were done in ANC clinic, where 460 (source population) pregnant mothers were divided for a sample size of 102 for the Chiro zonal hospital and 898 (source population) pregnant mothers were divided for a sample size of 200 for the Gelemso general hospital to get sample interval (kth value), which became 4 for both sites. Then, the first pregnant mother was randomly selected by the lottery method. Therefore, every 4th mother attending the clinics was enrolled in the study until the calculated sample size was achieved within 3 months of data collection. A total of 302 study participants were included in the study during the study period (Figure 1). Participants' identification number was used to avoid repetition.

### Data collection methods

A pretested and structured questionnaire adapted from the WHO checklist for the assessment of *hepatitis B infection* in antenatal patients (16) was used to collect information on sociodemographic information, circumcision, history of blood transfusion, risky sexual behavior, history of hospital admission and surgery, history of abortion, contact with HBV-infected individuals, and other factors. Data were collected by three trained midwives through a face-to-face interview. Data collectors introduced themselves and informed the participants of the purpose of the study. After the participants agreed to participate, they were asked to give written consent and were assigned a study identification number; then, the interview was held in a private room in the hospitals using a structured questionnaire.

### Blood sample collection and laboratory examination

At the time of arrival to the laboratory, all study participants' arms was cleaned with 70% denatured alcohol, and then approximately 3 ml of venous blood was collected using blood collection tubes following the standard operating procedure (SOP) by a trained laboratory technologist at the hospital laboratory. Blood collected by venipuncture was allowed to clot naturally. Serum was separated from whole blood cells and transferred to CryoTube and stored at −20°C. The serum sample was then transported to the Chiro Blood Bank Center Laboratory using a triple packing system with a vaccine carrier, where it was tested for HBsAg using an antibody sandwich enzyme-linked immunosorbent assay (ELISA) test kit (Human type, Germany) in accordance with the manufacturer's instructions. The test has a 99.9% specificity and 100% sensitivity. With monoclonal antibodies tailored to HBsAg, polystyrene microwell strips are used in the sandwich ELISA technique. A second antibody that was conjugated with horseradish peroxidase (HRP) and formed in the event that HBsAg was present in the sample, which is captured on the solid phase, was added to the microwells along with a patient sample. Since the color's intensity correlated with the antigen content of the sample, it was measured. Using a second AiDTM HBsAg ELISA, all reactive and some nonreactive results were confirmed.

### Operational definitions

The following operational definitions were used in this study:

**Prevalence**: Percentage of pregnant mothers that were affected with HBsAg during this study period at this study place.

**Abortion**: Spontaneous or deliberate ending of pregnancy before the fetus could be expected to survive.

**Female circumcision**: Comprises all procedures that involve partial or total removal of the external female genitalia traditionally.

**Piercing**: This is a type of body beautification where a sharp material punctures a hole in the body ears or nose parts where jewelry had been inserted.

**Tattoo**: Is any form of body art that is created when ink is inserted, using a needle or other sharp materials into the dermis layer of the skin.

**Tonsillectomy**: Traditional tonsillectomy: Complete or partial removal of tonsils traditionally.

**Chronic HBV infection**: Defined by the continued presence of HBsAg in the blood for longer than 6 months.

**Knowledge**: In this case, participants who scored 50% and above from all asked questions were labeled as have good knowledge and those who scored less than 50% were labeled as having poor knowledge on HBV infection and transmission (17).

### Quality assurance

The questionnaires were prepared in English and translated into Afaan Oromo language by an independent translator that could be understood by the study participants. Finally, the questionnaires were translated back to English to ensure their consistency with the previous initial version. Data collectors along with supervisors were trained on data collection procedures. The questionnaires were pretested on 15 pregnant mothers attending ANC clinics in the Chiro hospital to check their appropriateness and understandability by study participants before the actual data collection process to correct questionnaires. Supervisors and principal investigators checked the collected data for consistency and completeness. Codes were given to completed questionnaires. Oriented laboratory professionals collected blood specimens. Standardized procedures were followed during blood sample collection, storage, transportation, and analytical process. The ELISA test was performed by experienced laboratory personnel who were trained by Hema diagnosis laboratory with a Huma reader and Washer (Human, Germany). Quality control of serological tests, known positive and negative controls, was run in parallel with test samples. All laboratory procedures were carried out following SOP according to the manual instructions. The results were recorded with the patient's identification number, to avoid errors in the results of the test, and repeatedly checked before reporting.

### Data processing and analysis

Data were checked for completeness and consistency of the collected information, coded and entered into Epi-data version 3.1, and exported to Statistical Package for the Social Science (SPSS) version 22.0 software package for analysis. Descriptive statistical analysis was done using frequency tables, and proportions were used to summarize the data. Bivariate logistic regression analysis was carried out using cross-tabulation to see the association between HBsAg serostatus and independent variables while controlling for colinearity. Variables having a *P*-value less than 0.25 were included in the multiple logistic regression analysis at a 95% confidence interval. Then, multivariate logistic regression was done to control for possible confounders and identify the true effect of the selected predictor variables. The model adequacy was checked using the Hosmer–Lemeshow test to show the goodness of fitness. Finally, the strength of association between the outcome and predictor variables was assessed using an adjusted odd ratio (AOR) with a 95% confidence interval, and the significance of the association was declared at a *P*-value of less than 0.05.

### Ethical consideration

Ethical clearance was obtained from the Institutional Health Research Ethics Review Committee (IHRERC) of the Haramaya University College of Health and Medical Science. The ethical letter was submitted to the West Hararghe Zonal Health office and official permission was sought from the West Hararghe Zonal Health Office and Gelemso and Chiro public hospitals’ administration; then, support letter was obtained from hospitals’ administration. Written informed voluntary consent was obtained after informing the purpose and importance of the study to each participant. To ensure the confidentiality of the participant's information, a code was used instead of the name of the participant. Participants were interviewed alone to maintain privacy. Individual test results were communicated with the attending physician for further care as national protocol and health education were given to clients on preventive measures to be taken as well as to protect others.

## Results

### Sociodemographic characteristics of the study participants

A total of 300 pregnant mothers were included in this study with an overall response rate of 99%. The mean age of the study participants was 28.6 (SD ± 6.88) years and it ranged from 18 to 47 years. More than half of the participants (65.3%) were urban residents, and 79.3% were pregnant between 6 and 9 months. Nearly all of the participants, 298 (99.3%), were married and out of which 28 (9.3%) were polygamous. A total of 86 (28.7%) and 148 (49.3%) participants were not able to read and write and were merchants, respectively (Table 1).

**Table 1:** Sociodemographic characteristics of pregnant mothers attending ANC clinics in public hospitals in west Hararghe, eastern Ethiopia, from September to December 2020.

Variables	Characteristics	Frequency	Percent (%)
Age in years	≤29	126	42
	30–47	174	58
Residences	Urban	196	65.3
	Rural	104	34.7
Educational status	Not read and write	86	28.7
	Read and write	214	71.3
Religion	Muslim	149	49.7
	Orthodox	106	35.3
	Others	45	15.0
Ethnicity	Oromo	198	66.0
	Amhara	71	23.7
	Others	31	10.3
Marital status	Married (mono)	270	90.0
	Married (poly)	28	9.3
	Single	2	0.7
Occupational status	Government employee	29	9.7
	Private employee	4	1.3
	Merchant	148	49.3
	Housewife	119	39.7

ANC, antenatal care.

### Pregnancy-related, cultural, and clinical characteristics

The majority of study participants (81.7%) had more than three children, had a history of body piercing (87%), and had previously undergone an abortion at a health facility [53 (74%)] and at home [19 (26%)], of which 5 (31%) at home and 11 (69%) in a health facility were HBsAg positive. More than half of the study participants had a history of female genital mutilation (FGM) (60.3%). Of the participants, 65% did not practice home delivery, and 56% had a history of health facility admission. A total of A total of 111 (37%), 50 (17%), 64 (21%), 68 (22.7%), and 125 (41.7%) had an experience of a tattoo, blood transfusion, surgical procedures, dental procedures, and tonsillectomy, respectively. About 49 (16.7%) study participants had multiple sexual partners (Table 2).

**Table 2:** Pregnancy, cultural and clinical related characteristics of pregnant mothers attending ANC clinics in public hospitals in west Hararghe, Eastern Ethiopia, from September to December 2020.

Variables	Characteristics	Frequency	Percent (%)
Parity	≤3	246	82
	>3	54	18
Gestational age in months	1–3	9	3.0
	4–5	53	17.7
	6–9	238	79.3
History of home delivery by TBA	Yes	103	34.3
	No	197	65.7
History of abortion	Yes	72	24
	No	228	76
FGM history	Yes	181	60.3
	No	119	39.7
Tattooing	Yes	111	37
	No	189	63
History of body piercing	Yes	261	87
	No	39	13
History of health facility admission	Yes	168	56
	No	132	44
History of blood transfusion	Yes	50	17
	No	250	83
History of surgical procedures	Yes	64	21
	No	236	79
Dental extraction	Yes	68	22.7
	No	232	77.3
History of tonsillectomy	Yes	125	41.7
	No	175	58.3
History of sexually transmitted infection	Yes	77	25.7
	No	223	74.3
History of multiple sexual partners	Yes	49	16.3
	No	251	83.7
History of jaundice	Yes	61	20.3
	No	239	79.7
History of contact with jaundice person	Yes	34	11.3
	No	266	88.7
HIV test result	Positive	14	4.7
	Negative	286	95.3

TBA, traditional birth attendants; FGM, female genital mutilation; HIV, human immunodeficiency virus.

### Knowledge about HBV

Out of the 300 study participants, 239 (79.7%) had poor knowledge. Of the participants, 35% and 38% had heard of a disease termed hepatitis B as a viral disease and affected liver function, respectively. Only 25% knew nausea, vomiting, and loss of appetite are common symptoms of *hepatitis B*. About 26%, 16%, and 33% of the study participants said there is no symptom of hepatitis B virus infection, HBV transmission can occur from mother to child during pregnancy, and HBV has a vaccine, respectively (Table 3).

**Table 3:** Knowledge about *HBV* among pregnant mothers attending routine ANC clinics in public hospitals in west Hararghe, eastern Ethiopia, from September to December 2020.

Variables	Characteristics	The result for HBV		
		Positive, *n* (%)	Negative, *n* (%)	Total, *n* (%)
Hepatitis B is a viral disease	Yes	7 (7)	97 (93)	104 (35)
	No	17 (9)	179 (91)	196 (65)
Hepatitis B can affect liver function	Yes	12 (11)	102 (89)	114 (38)
	No	12 (7)	174 (94)	186 (62)
Hepatitis B can cause liver cancer	Yes	6 (26)	17 (74)	23 (8)
	No	18 (7)	259 (94)	277 (92)
Hepatitis B can affect any age group	Yes	8 (12)	61 (88)	69 (23)
	No	16 (7)	215 (93)	231 (77)
Jaundice is the common symptoms of hepatitis B	Yes	12 (10)	114 (90)	126 (42)
	No	12 (7)	162 (93)	174 (58)
Nausea, vomiting and loss of appetite are common symptom of hepatitis B	Yes	10 (13)	66 (87)	76 (25)
	No	14 (6)	210 (94)	224 (75)
We can't see symptoms of the hepatitis B in some of the patients	Yes	4 (5)	75 (95)	79 (26)
	No	20 (9)	201 (91)	221 (74)
Hepatitis B can be transmitted by un sterilized syringe needle and surgical instrument	Yes	4 (10)	36 (90)	40 (13)
	No	20 (7.7)	240 (92)	260 (87)
Hepatitis B can be transmitted by contaminated blood and blood product	Yes	6 (13)	40 (87)	46 (15)
	No	18 (7)	236 (93)	254 (85)
Hepatitis B can be transmitted by blades of the tattoo/ear or nose pierces	Yes	4 (13)	26 (87)	30 (10)
	No	20 (7)	250 (93)	270 (90)
Hepatitis B can be transmitted by unsafe sex	Yes	5 (12)	36 (88)	41 (14)
	No	19 (7)	240 (93)	259 (86)
Hepatitis B can be transmitted by mother to child	Yes	5 (10)	42 (89)	47 (16)
	No	19 (8)	234 (92)	253 (84)
Hepatitis B can be transmitted by contaminated water/food prepared by person suffering with the infections	Yes	5 (10)	46 (90)	51 (17)
	No	19 (8)	230 (92)	249 (83)
Hepatitis B curable/treatment available	Yes	13 (12)	94 (88)	107 (36)
	No	11 (6)	182 (94)	193 (64)
Hepatitis B can be self-cured by body	Yes	9 (15)	51 (85)	60 (20)
	No	15 (6)	225 (94)	240 (80)
Vaccination is available for hepatitis B	Yes	11 (11)	89 (89)	100 (33)
	No	13 (7)	187 (94)	200 (67)
Specific diet is required for treatment of hepatitis B	Yes	5 (20)	20 (80)	25 (8)
	No	19 (7)	256 (93)	275 (92)
Knowledge				
Good ≥50%	Yes	10 (16.4)	51 (83.6)	61 (20.3)
Poor <50%	No	14 (5.9)	225 (94.1)	239 (79.7)

HBV, hepatitis B virus; ANC, antenatal care.

### Seroprevalence of hepatitis B virus infection

The overall prevalence of HBV among pregnant mothers was 8% (24/300) (95% CI: 5.3–11.0). Among those HBV-seropositive pregnant mothers, 15 (62.5%) and 9 (37.5%) were from the Gelemso and Chiro Hospitals, respectively. The proportion of *HBV* was 9% among the 31–49 year age groups. Additionally, as one's level of education increases, the prevalence of HBV decreases.

### Factors associated with hepatitis B virus infection

In bivariate analysis, age, residence, educational status, history of home delivery, FGM, parity, history of abortion, tattooing, history of admission to the health facility, history of blood transfusion, history of surgical procedure, history of dental extraction, history of tonsillectomy, history of cesarean section, history of sexually transmitted diseases, history of multiple sexual partners, history of jaundice, history of contact with liver disease patient, and knowledge about HBV were candidate variables for multivariate analysis (Table 4).

**Table 4:** Bivariate analysis of factors associated with hepatitis B virus infection among pregnant mothers attending ANC clinics in public hospitals in west Hararghe, eastern Ethiopia, from September to December 2020.

Variables	Characteristics	HBsAg status		COR (95% CI)	*P*-value
		Positive, *n* (%)	Negative, *n* (%)		
Age in years	18–30	9 (7)	117 (93)	1	
	31–49	15 (9)	159 (91)	1.2 (0.5–2.9)	0.64
Gestational age in months	1–3	1 (11.1)	8 (88.9)	1	
	4–5	4 (7.5)	49 (92.5)	0.7 (0.1–6.6)	0.72
	6–9	19 (8.0)	219 (92.0)	.7 (0.1–5.9)	0.74
Residence	Urban	10 (5.1)	186 (94.9)	1	
	Rural	9 (13.5)	90 (86.5)	2.9 (1.2–6.8)	0.01^a^
Educational status	Not read	12 (14.0)	74 (86.0)	2.7 (1.2–6.3)	0.02^a^
	Write and read	12 (5.6)	202 (94.4)	1	
History of home delivery (TBA)	Yes	15 (14.6)	88 (85.4)	1	
	No	9 (4.6)	188 (95.4)	3.6 (1.5–8.5)	0.004^a^
Parity	≤3	18 (7.3)	228 (92.7)	1	
	>3	6 (11.7)	48 (88.9)	1.5 (0.6–4.2)	0.36
History of abortion	Yes	16 (22.2)	56 (77.8)	1	
	No	8 (3.5)	220 (96.5)	7.9 (3.2–19.3)	0.00^a^
FGM	Yes	21 (11.6)	160 (88.4)	1	
	No	3 (2.5)	116 (97.5)	5.1 (1.5–17.4)	0.01^a^
Tattoo	Yes	17 (15.3)	94 (84.7)	1	
	No	7 (3.7)	182 (96.3)	4.7 (1.9–11.7)	0.00^a^
History of health facility admission	Yes	21 (12.5)	147 (87.5)	1	
	No	3 (2.3)	129 (97.7)	6.1 (1.8–21.1)	0.01^a^
History of blood transfusion	Yes	14 (28.0)	36 (72.0)	1	
	No	10 (8.3)	111 (91.7)	4.3 (1.8–10.6)	0.00^a^
History of surgical procedure	Yes	17 (26.6)	47 (73.4)	1	
	No	7 (6.5)	100 (93.5)	5.2 (2.0–13.3)	0.00^a^
Dental extraction	Yes	18 (26.5)	50 (73.5)	1	
	No	6 (2.6)	226 (97.4)	13.6 (5.1–35.9)	0.00^a^
History of tonsillectomy	Yes	19 (15.2)	106 (84.8)	1	
	No	5 (2.9)	170 (97.1)	6.1 (2.2–16.8)	0.00^a^
History of CS	Yes	13 (25.5)	38 (74.5)	1	
	No	11 (4.4)	238 (95.6)	7.4 (3.1–17.7)	0.00^a^
History of STI	Yes	14 (18.2)	63 (81.8)	1	
	No	10 (4.5)	213 (95.5)	4.7 (2.0–11.2)	0.00^a^
History of multiple sexual partners	Yes	16 (32.7)	33 (67.3)	1	
	No	8 (3.2)	243 (96.8)	14.7 (5.9–37.1)	0.00^a^
History of jaundice	Yes	14 (23)	47 (77)	1	
	No	10 (4.2)	229 (95.8)	6.8 (2.8–16.3)	0.00^a^
Previous history of contact with a liver disease patient	Yes	9 (26.5)	25 (73.5)	1	
	No	15 (5.6)	251 (94.4)	6.0 (2.4–15.1)	0.00^a^
Screened for HBV	Yes	4 (12.1)	29 (87.9)	1	
	No	20 (7.5)	247 (92.5)	1.7 (0.5–5.3)	0.36
HIV test result	Positive	4 (28.6)	10 (71.4)	1	
	Negative	20 (7.0)	266 (93.0)	5.3 (1.5–18.5)	0.01^a^
Knowledge about HBV	≥50	10 (16.4)	51 (83.6)	1	
	<50	14 (5.9)	225 (94.1)	3.2 (1.3–7.5)	0.01^a^

COR, crude odd ratio; CI, confidence interval; HBV, hepatitis B virus; 1, reference category; ANC, antenatal care; TBA, human immunodeficiency virus; FGM, female genital mutilation; HIV, human immunodeficiency virus; STI, sexually transmitted infection; CS, caesarean section.

^a^ Variables selected for multivariate analysis (*P*-value <0.25 and fit model test).

In multivariable analysis, those pregnant mothers who had a tattoo, history of tonsillectomy, history of multiple sexual partners, and previous contact with liver disease patients were identified to have factors associated with HBV infection.

Pregnant mothers who had a tattoo had 4.3 times the odds of being HBsAg positive compared to those who did not have tattoos (adjusted odds ratio = 4.3; 95% CI: 1.1–17.0). Similarly, pregnant mothers who had a history of tonsillectomy had 5.7 times the odds of being HBsAg positive compared those who did not have a history of tonsillectomy (adjusted odds ratio = 5.7, 95% CI: 1.3–23.9). Mothers who had a history of contact with liver disease patients had 5.6 times the odds of being HBsAg positive compared to their counterparts (adjusted odds ratio = 5.6; 95% CI: 1.2–25.7). Pregnant mothers who had a history of multiple sexual partners had 10 times the odds of being HBsAg positive compared to those who did not have history of multiple sexual partners (adjusted odds ratio = 10.8; 95% CI: 2.5–45.9) (Table 5).

**Table 5:** Multivariable logistic regression analysis for factors associated with hepatitis B virus infection among pregnant mothers attending ANC clinics in public hospitals in west Hararghe, eastern Ethiopia, from September to December 2020.

Variables	Characteristics	HBsAg status		COR (95% CI)	AOR (95% CI)
		Positive, *n* (%)	Negative, *n* (%)		
Educational status	Not read	12 (14.0)	74 (86.0)	2.7 (1.2–6.3)	2.4 (0.6–9.8)
	Write and read	12 (5.6)	202 (94.4)	1	1
Abortion history	Yes	16 (22.2)	56 (77.8)	7.9 (3.2–19.3)	2.3 (0.6–8.8)
	No	8 (3.5)	220 (96.5)	1	1
Tattoo	Yes	17 (15.3)	94 (84.7)	4.7 (1.9–11.7)	4.3 (1.1–17.0)
	No	7 (3.7)	182 (96.3)	1	1
Blood transfusion	Yes	14 (28.0)	36 (72.0)	4.3 (1.8–10.6)	1.4 (0.4–5.4)
	No	10 (8.3)	111 (91.7)	1	1
Surgical procedure	Yes	17 (26.6)	47 (73.4)	5.2 (2.0–13.3)	4.5 (0.9–20.5)
	No	7 (6.5)	100 (93.5)	1	1
Dental extraction	Yes	18 (26.5)	50 (73.5)	13.6 (5.1–35.9)	3.5 (0.9–13.3)
	No	6 (2.6)	226 (97.4)	1	1
Tonsillectomy	Yes	19 (15.2)	106 (84.8)	6.1 (2.2–16.8)	5.7 (1.3–23.9)
	No	5 (2.9)	170 (97.1)	1	1
History of CS procedure	Yes	13 (25.5)	38 (74.5)	7.4 (3.1–17.7)	0.54 (0.1–2.4)
	No	11 (4.4)	238 (95.6)	1	1
History of multiple sexual partners	Yes	16 (32.7)	33 (67.3)	14.7 (5.9–37.1)	10.8 (2.5–45.9)
	No	8 (3.2)	243 (96.8)	1	1
Previous history of contact with liver disease patient	Yes	9 (26.5)	25 (73.5)	6.0 (2.4–15.2)	5.6 (1.2–25.7)
	No	15 (5.6)	251 (94.4)	1	1

ANC, antenatal care; AOR, adjusted odd ratio; COR, crude odds ratio; CI, confidence interval.

## Discussion

The overall prevalence of hepatitis B virus among pregnant mothers attending routine ANC clinics was 8% (95% CI: 5.3–11.0); this finding was classified as high according to the WHO classification. History of a tattoo, history of tonsillectomy, experiencing multiple sexual partners, and history of contact with HBV-infected patients were factors found to be associated with hepatitis B virus infection among pregnant mothers.

The current study's findings were in agreement with those of a study carried out in various countries (Table 6).

**Table 6:** Prevalence of *Hepatitis B* infection among pregnant mothers conducted in different countries.

Ethiopia	Other countries
Dire Dawa: 8.4% (18),	Uganda: 11.8% (19)
Hawassa: 7.8% (20)	Yemen: 10.8% (21)
Gambella: 7.9% (22)	Southwest Nigeria: 10.5% (23)
Deder Hospital: 6.9% (15)	Cameroon: 9.7% (17)
Bishoftu Hospital: 5.4% (24)	Australia: 13.8% (25)
Southern Ethiopia Yirgalem hospital: 7.2% (26); Bahir Dar city 6.6% (27)	

However, the current study finding is higher than the study conducted at the Arba Minch Hospital in southern Ethiopia (4.3%0 (28), Gandhi hospital in Addis Ababa (2.3%) (29), Dawuro hospital in southern Ethiopia (3.5%) (30), and Felegehiwot hospital in Bahir Dar (4.7%) (31), as well as in Kenya (3.8%) (12), Tanzania (3.9%) (32), Rwanda (3.7%) (33), and Turkey (2.1%) (34). This variation might be due to differences in the sociodemographic characteristics of study participants, geographical area, health policy implemented on hepatitis prevention strategies, differences in cultural practices of community, sexual behaviors as well as differences in sampling method, small sample size, and laboratory test methods employed to detect HBsAg. Additionally, it could be due to poor adherence with the national immunization program, known as the expanded program on immunization (EPI) in Ethiopia (35).

According to this study, pregnant mothers who had tattoos on any part of their bodies were more likely to acquire hepatitis B infection than their counterparts. This finding is comparable to research done in Ethiopia, Bahir Dar (27), southwestern Bench-Maji (36), and Debra-Tabor (37), and in Antioch of Turkey (34) and China (38). This could be attributed to the fact that tattoos are performed using non-sterile equipment. This suggests that the spread of HBV in the study area may be facilitated by the sharing of hazardous materials for customary practice. Tonsillectomy and tattooing are traditional practices in this study area. Hepatitis B virus can remain alive and infectious on surfaces of contaminated materials for at least 7 days, such as blood (5).

Pregnant mothers who had a history of tonsillectomy were at higher risk of HBV infection compared to those who did not. A similar finding was reported in a study conducted in Dawuro in southern Ethiopia (28), Uganda (39), and Nigeria (40). This finding may be explained as most of the time tonsillectomy was practiced with a traditional practitioner possibly by using repeatedly unsterilized instruments, which might increase the probability of transmission of HBV infection in the community.

Hepatitis B virus infection is a sexually transmitted disease and the transmission increases with the duration of sexual activity and the number of sexual partners exposed (4). In the current study, pregnant mothers having a history of multiple partners were more likely to be infected with HBV. This is in agreement with the findings from other parts of Deder (15), Dessie (41), Dawuro in southern Ethiopia (40), Yirgalem (26), Arba Minch (28), Bishoftu (42), Felegehiwot in Bahir Dar (42), and Congo (43) and southern Uganda (44).

Moreover, our study revealed that pregnant mothers who had a history of contact with a jaundiced patient had a higher chance of acquiring hepatitis B infection. This finding is supported by the study conducted at the Attat hospital in southern Ethiopia (45) and Mizan-Tepi (46). In addition to the difference in behavioral and cultural practice, exposure to the chronic carrier has a wide chance of contact with body fluids. Our findings are in accordance with other studies reported, which indicate that the risk of HBV transmission is high in people who are in contact with chronically infected HBV participants (47).

Residence, abortion, and blood transfusion did not show a significant association with HBV infection. The residence of participants and educational status were in agreement with study findings in Hawassa (20) and Arba Minch (28), which is opposite to a study in Dessie (48). History of having an abortion failed to indicate a significant association among pregnant mothers as it is in line with other studies conducted in Bahir Dar (27), contrary to studies in Deder (15) and Gambella (49). History of blood and blood product transfusion failed to show any evidence of association of virus transmission, which was in agreement with studies of HBV infection in pregnant mothers in Nairobi, Kenya (32), Kinshasa, Tanzania (32), and Mulago hospital in Uganda(50), but it contradicted other studies that indicated evidence of blood transfusion was significantly associated with virus transmission in Bishoftu in Ethiopia (24), and Arba Minch in southern Ethiopia (28). The possible explanation may be the variation in study area and period, sample size, safety precaution being taken, and proper screening for virus in a regularly carried out manner currently before blood donation in the study area.

Pregnant mothers with a history of surgical procedures failed to show *hepatitis B* infection association with virus transmission supported by studies conducted in Hawassa (20), Gambella hospital (50), Dawuro in southern Ethiopia (20, 30). and Arba Minch (28), in contrary to study findings in Addis Ababa (42) and Harar in Ethiopia (51), Deder in eastern Ethiopia (15), and Bahir Dar in northwest Ethiopia (27). The difference may be attributed to variation, the use of standard procedures, and disinfected instruments by a health professional.

The current study showed that about 78% of study participants had poor knowledge (scored less than 50%) regarding HBV infection, mode of transmission, and other. Only about 84 (28%), 43 (14%), and 73 (24%) study participants responded positively about HBV infection, transmission, and prevention-related knowledge, respectively. It has been documented that community awareness of the transmission of hepatitis B is low in Ethiopia (52). A similar low knowledge was observed in a study conducted in Cameron (53). This may be related to the type of occupation due to nonadherence to guidelines on infection control, the use of non-disposable or reusable equipment, and the lack of adequate sterilization techniques as it is mostly with those who have knowledge better than those who want to get service.

## Strengths and limitations of study

One of the strengths of this study was the use of the ELISA method for laboratory examination, which is uncommon in Ethiopia to test for HBV. Another strength was the identification of potential associated risk factors using quality-assured data obtained by trained personnel using the pretested structured questionnaire. However; laboratory tests for serological markers such as hepatitis B core antibody, hepatitis B surface antibody, total hepatitis B core antibody, and IgM antibody to hepatitis B core antigen, which were necessary to diagnose HBV infection and to identify the stage of infection, were not performed due to resource limitation. Additionally, due to the lack of resources, HBsAg-positive pregnant women were not tested for HBeAg or viral load, making it impossible for this study to determine the extent of perinatal transmission of HBV. The study was also conducted in antenatal clinics, which can limit results representative of the entire population, and information obtained from mothers could be subjected to responder bias.

## Conclusion and recommendations

The prevalence of *HBV* infection among pregnant mothers attending antenatal care clinics in west Hararghe public hospitals was high according to the WHO classification, suggesting that *HBV* is an important public health issue in the study area. History of having a tonsillectomy, tattooing, having multiple sexual partners, and having contact with jaundiced patients were identified as factors associated with *hepatitis B* virus infection.

In order to reduce transmissions, it is recommended that the government should start HBV vaccination campaigns, begin with mass vaccination, and implement a national immunization program. As soon as possible after birth, ideally within 24 h, all newborns should receive hepatitis B vaccines. Additionally, to reduce the risk of transmission from mother to child, all pregnant women with HBV infection should receive HBsAg testing and take antiviral prophylaxis. The health bureau and medical professionals should also educate expectant mothers more about preventing and controlling HBV infection.

## Data Availability

The original contributions presented in the study are included in the article/Supplementary Material, further inquiries can be directed to the corresponding author.
